# A Novel Frontier in Gut–Brain Axis Research: The Transplantation of Fecal Microbiota in Neurodegenerative Disorders

**DOI:** 10.3390/biomedicines13040915

**Published:** 2025-04-09

**Authors:** Majid Eslami, Zarifeh Adampour, Bahram Fadaee Dowlat, Shayan Yaghmayee, Faezeh Motallebi Tabaei, Valentyn Oksenych, Ramtin Naderian

**Affiliations:** 1 Cancer Research Center, Semnan University of Medical Sciences, Semnan 35147-99442, Iran; m.eslami@semums.ac.ir; 2 Department of Bacteriology and Virology, Faculty of Medicine, Semnan University of Medical Sciences, Semnan 35147-99442, Iran; 3 Institute of Science, Biotechnology and Biosafety Department, Eskishehir Osmangazi University, Eskishehir 26040, Türkiye; zarifeh.adampour@yahoo.com; 4 School of Medicine, Iran University of Medical Sciences, Tehran 14496-14535, Iran; 5 Nervous System Stem Cells Research Center, Semnan University of Medical Sciences, Semnan 35147-99442, Iran; 6 Department of Medical Microbiology, Faculty of Medicine, Golestan University of Medical Sciences, Gorgan 49189-36316, Iran; 7 Faculty of Medicine, University of Bergen, 5020 Bergen, Norway; 8 Clinical Research Development Unit, Kowsar Educational, Research and Therapeutic Hospital, Semnan University of Medical Sciences, Semnan 35147-99442, Iran

**Keywords:** fecal microbiota transplantation, gut–brain axis, neurodegenerative diseases, Parkinson’s disease, Alzheimer’s disease

## Abstract

The gut–brain axis (GBA) represents a sophisticated bidirectional communication system connecting the central nervous system (CNS) and the gastrointestinal (GI) tract. This interplay occurs primarily through neuronal, immune, and metabolic pathways. Dysbiosis in gut microbiota has been associated with multiple neurodegenerative diseases, such as Parkinson’s disease (PD), Alzheimer’s disease (AD), multiple sclerosis (MS), and amyotrophic lateral sclerosis (ALS). In recent years, fecal microbiota transplantation (FMT) has gained attention as an innovative therapeutic approach, aiming to restore microbial balance in the gut while influencing neuroinflammatory and neurodegenerative pathways. This review explores the mechanisms by which FMT impacts the gut–brain axis. Key areas of focus include its ability to reduce neuroinflammation, strengthen gut barrier integrity, regulate neurotransmitter production, and reinstate microbial diversity. Both preclinical and clinical studies indicate that FMT can alleviate motor and cognitive deficits in PD and AD, lower neuroinflammatory markers in MS, and enhance respiratory and neuromuscular functions in ALS. Despite these findings, several challenges remain, including donor selection complexities, uncertainties about long-term safety, and inconsistencies in clinical outcomes. Innovations such as synthetic microbial communities, engineered probiotics, and AI-driven analysis of the microbiome hold the potential to improve the precision and effectiveness of FMT in managing neurodegenerative conditions. Although FMT presents considerable promise as a therapeutic development, its widespread application for neurodegenerative diseases requires thorough validation through well-designed, large-scale clinical trials. It is essential to establish standardized protocols, refine donor selection processes, and deepen our understanding of the molecular mechanisms behind its efficacy.

## 1. Introduction

### 1.1. Mechanisms and Implications of the Gut–Brain Axis

The gut–brain axis (GBA) represents a bidirectional network, linking the CNS to the GI system [1]. This intricate system involves neuronal, hormonal, and immune pathways, which have a pivotal role in the homeostasis of both areas. The axis includes interaction between the enteric nervous system, gut microbiota, and CNS, with particularly identified pathways encompassing the vagus nerve, spinal autonomic pathway, hormones, chemical metabolites such as cytokines, and neurotransmitters [2]. Through these, the GBA plays a critical role in CNS-related performance, including memory, cognitive function, mood behavior, etc. In the following, we will discuss some identified linking pathways between gut microbiota and the CNS.

### 1.2. Neuronal Pathway

The neuronal pathway is primarily established by the vagus nerve. The vagus nerve is the 10th cranial nerve, containing various types of fibers, including both sensory (80% of fibers) and motor (20% of fibers) ones [3]. The motor fiber mostly originates from the dorsal nucleus of the vagus nerve and the ambiguous nucleus, transmitting signals into smooth muscle and striated muscle, respectively. Sensory fibers (also known as afferent fibers) arise from the target organs (e.g., gastrointestinal tissue) and relay signals into the sensory nucleus of the vagus nerves (e.g., the nucleus of the solitary tract (NTS)). Afferent fibers do not cross from the epithelial layer [4]. Some afferent fibers end in the muscular layer, which is responsible for sensing mechanical stimulation; the last fibers terminate in the mucosal and submucosal layer, forming a synaptic-like structure with two group cells: epithelial cells and EEC (1% of intestinal epithelial cells). The enteroendocrine cells (EEC) communicate with afferent fibers through a neuropool-like extension [5]. EECs stimulate the vagus nerve by releasing 5-hydroxytryptamine 3 (5-HT3), cholecystokinin (CCK), peptide YY (PYY), glucagon-like peptide-1 (GLP-1), etc. Microbiota can influence afferent terminals in two ways: (1) directly, by releasing substances such as short-chain fatty acids (SCFAs), 5-HT3, and 5-HT4; and (2) indirectly, by EECs being activated by bacteria and then stimulating fibers [6]. In that case, microbiota-derived SCFA induces tryptophan-hydroxylase (TH) expression in Enterochromaffin-like (ECL) cells and releases 5-HT. ENS is engaged in the gut–brain connection in other ways such as autonomic patterns. It also regulates gut motility, secretion, and local reflexes while influencing brain functions via gut-derived neurotransmitters. The insights into olanzapine-induced lipid disturbances through the gut microbiota–brain axis contribute to the growing understanding of gut–brain interactions, especially in the context of neurodegenerative disorders. This axis plays a critical role in regulating lipid metabolism, neurotransmitter synthesis, and inflammatory processes. Disruptions within it have been linked to conditions like PD and AD. The vagus nerve, a pivotal component of this system, appears to mediate the effects of olanzapine on lipid metabolism, echoing its involvement in neuroinflammatory pathways influenced by gut microbiota. These findings highlight the potential of microbiota-targeted therapies to reduce both metabolic and neurological side effects associated with olanzapine use [7].

### 1.3. Immune Pathway

Gut microbiota regulate the inflammatory or anti-inflammatory states of the brain. They can increase the production of inflammatory cytokines including interleukin 6 (IL-6), tumor necrosis factor-alpha (TNF-α), and IL-1β, which cross the blood–brain barrier (BBB) and influence the brain tissue [8,9]. This highlights the crucial role of understanding microbial recovery and metabolic balance in achieving precise enumeration and efficient microbial management for food safety [10]. This approach proposes that managing the gut microbiota might be an effective strategy for lowering uric acid levels and mitigating related complications, including kidney damage [11]. Additionally, microbiota-derived metabolites influence immunological cells like microglia, resulting in their activation or inhibition [12]. SCFAs exhibit other beneficial functions; in the gut lumen, they decrease the activation of polymorphonuclear neutrophils (PMNs) and neutrophils, leading to a decrease in immune processes like inflammation [13,14]. Furthermore, SCFAs in the bloodstream can cross the BBB, where they act on microglia to promote oligodendrocyte maturation. This process enhances the myelination of CNC neurons, offering a neuroprotective effect [15]. The lever between inflammatory and anti-inflammatory status in CNS plays the most significant role in the development of neurodegenerative diseases, such as AD and PD [16]. It has also been demonstrated that dysbiosis leads to alternation in immune cell trafficking, including Treg and TH17 [17]. It diminishes the Treg population and impairs the anti-inflammatory state, while TH17 increases. The enhancement of the latter may lead to an autoimmune system like MS [18]. Beyond that, It has been indicated that the dysbiosis of gut microbiota causes a “leaky gut”, allowing pathogens to enter the circulation; this may lead to neuroinflammation status in neurological disorders like ASD [12].

### 1.4. Molecular Pathway

Butyrate and acetate, major metabolites of gut microbiota, play diverse roles in microbial communities [19,20]. SCFAs regulate microglial activation, promoting an anti-inflammatory state and enhancing CNS homeostasis [21]. The findings suggest that adjusting the gut microbiota composition and minimizing inflammation are essential strategies for safeguarding against metabolic and liver-related disorders [22]. They also are involved in the expression regulation of BDNF, which is a critical factor in neuronal plasticity [23]. They also can increase tight junction proteins [24], resulting in the enhancement of BBB integrity as a natural barrier against toxic metabolites [25]. Gut microbiota also influence kynurenine pathways of tryptophan metabolism, resulting in the production of neuroactive metabolites such as kynurenine and quinolinic acid [26]. The dysregulation of this pathway results in the accumulation of neurotoxic agents (e.g., quinolinic acid) and a reduction in the level of neuroprotective metabolites (kynurenic acid), contributing to mood disorders and neurodegeneration [27]. These findings indicate that changes in bile acid composition and gut microbiota could play a role in metabolic dysregulation, influencing liver function and promoting systemic inflammation [28]. Furthermore, gut microbiota are responsible for the production of various types of neurotransmitters, including serotonin, dopamine, gamma-aminobutyric acid (GABA), etc. [29,30,31]. It is also indicated that the conversion of primary bile acid to secondary bile acid, which is performed by microbiota, is essential in CNS regulation [32,33]. Secondary bile acid interacts with CNS receptors, encompassing farnesoid X receptor (FXR) and Takeda G-protein receptor 5 (TGR5) [34,35]; this interaction plays a pivotal role in neuronal signaling, energy metabolism, and inflammatory pathways in the brain [36].

## 2. FMT in Neurodegenerative Disorders

### 2.1. Gut Dysbiosis and Neurodegenerative Diseases

As previously mentioned, microbiota play a significant role in the regulation of brain functions. Due to this, alternation in the microbiota composition, known as dysbiosis, can lead to significant impairment in brain functions, causing the development of certain neurological diseases [37,38]. This indicates that modifying dietary components and the composition of gut microbiota can significantly contribute to enhancing metabolic efficiency and promoting the overall health of livestock [39]. This is confirmed by the findings of previous studies, which reveal that there is a remarkable change in the microbiota composition of subjects with neurodegenerative disorders, both in animal and clinical models. There are abundant proposed mechanisms under investigation that are not completely established. Alterations in bacterial composition present as increases in the inflammatory genera while diminishing anti-inflammatory ones, which may lead to the development of inflammation status in brain tissue [40]. This effect can be led by the activation of the master inflammatory cell in brain tissue and microglia. The study highlights the vital role of transforming growth factor beta-1 (TGF-β1) in managing neuroinflammatory responses and supporting the recovery of neural functions, offering valuable insights into potential therapeutic approaches for neurodegenerative diseases. Furthermore, it explores the therapeutic promise of *Lactobacillus paracasei* GY-1 in regulating gut microbiota and reducing inflammatory reactions, which could prove beneficial for conditions linked to metabolic and immune dysregulation [41,42]. Specific metabolites of inflammatory bacteria can stimulate microglia to initiate inflammation, resulting in neurodegeneration [43,44]. Similarly, Apelin-13 has demonstrated the ability to combat neuroinflammation associated with AD by lowering IL-1β and TNF-α levels and promoting brain-derived neurotrophic factor/tropomyosin-related kinase B receptor (BDNF/TrkB) signaling. This highlights its potential as a therapeutic approach to alleviate cognitive decline [45]. Gut microbiota and immune system interplay are crucial in managing metabolic functions and controlling inflammation, influencing both gastrointestinal health and systemic disorders [46,47]. This highlights the importance of targeting both amyloid-beta (Aβ) buildup and metabolic disturbances, such as insulin resistance, to develop more effective therapeutic strategies [48]. Dysbiosis can also lead to a leaky gut, which causes the elevation of toxic components in blood like lipopolysaccharides (LPS) [49]. LPS can stimulate TLR, resulting in the elevation of inflammatory cytokines including IL-1β, IL-6, and TNF-α, which can cross the BBB and contribute to neurodegeneration [50].

### 2.2. Preclinical and Clinical Evidence

Due to the significant role of gut microbiota in brain regulation, FMT may represent a novel therapeutic method in such disease, aiming at managing or even curing the disease. In the following, we investigated evidence as to the efficiency of FMT in treating neurological diseases. FMT has gained attention as an innovative therapeutic approach for neurodegenerative diseases, primarily through its ability to modulate gut microbiota and restore homeostasis in the gut–brain axis. Both preclinical and clinical research highlight its potential in managing conditions such as PD, AD, and MS. In the PD, FMT has been shown to alleviate motor dysfunction, reduce neuroinflammation, and regulate microbial populations such as *Akkermansia* and *Desulfovibrio*. Mechanistically, it suppresses pro-inflammatory signaling pathways, including toll-like receptor 4 (TLR4)/myeloid differentiation primary response 88 (MyD88)/nuclear factor-kappa B (NF-kB), while enhancing dopamine and serotonin levels within the substantia nigra. Clinical trials have observed improvements in gastrointestinal health, motor function, and microbial diversity among PD patients, though the durability of these benefits over time remains under investigation. For AD, FMT has demonstrated potential benefits such as reducing amyloid-β plaques, improving cognitive performance, and modulating neuroinflammatory responses. Transplants from younger donors tend to offer additional advantages, likely due to their more diverse and neuroprotective microbiota. Furthermore, FMT has been linked to alterations in the gut metabolome, including elevated levels of SCFAs and decreased inflammatory cytokines. These changes are believed to support synaptic plasticity and contribute to neuroprotection. A clinical study was conducted involving AD patients to evaluate the potential of FMT as a therapeutic approach for AD. Mild cognitive impairment patients show improved cognitive scores and improved daily abilities after FMT. Additionally, severe cognitive impairment patients showed no worsening in cognitive scores. Due to further investigation, it had been observed that FMT increased the diversity of gut microbiota, as confirmed by many other studies. The abundance of anti-inflammatory species *Prevotella* and *Eggerthellaceae* was increased, while the level of pro-inflammatory ones like *Lachnospira* was reduced. The study reported elevated levels of beneficial anti-inflammatory metabolites, such as deoxycholic acid and 3β, 12α-dihydroxy-5α-cholanoic acid, and significantly diminished levels of inflammatory metabolites like bilirubin [51,52,53].

**Figure 1:**
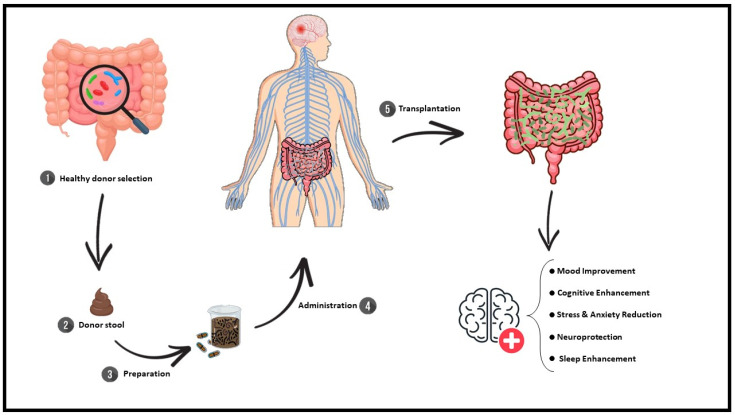
FMT transplantation. Schematic representation of the FMT process. (1) A healthy donor is selected based on specific criteria to ensure microbiome quality; (2) donor stool is collected; (3) the stool undergoes preparation, including processing into capsules or suspension; (4) the prepared microbiota are administered to the recipient; (5) following transplantation, gut microbiota balance is restored, leading to potential neurological and psychological benefits, including mood improvement, cognitive enhancement, stress and anxiety reduction, neuroprotection, and sleep enhancement.

In MS, FMT has proven promising in preclinical studies by restoring gut microbial balance, reducing microglial activation, and strengthening BBB integrity. Research using experimental autoimmune encephalomyelitis (EAE) models has demonstrated increases in SCFA-producing bacteria, decreased neuroinflammation, and lower levels of axonal damage. Although human studies are limited, preliminary findings suggest that FMT may improve gut barrier function and modulate immune responses beneficially in individuals with MS. FMT in neurodegenerative diseases is shown in Table 1 and Figure 1.

The findings highlight significant trends, challenges, and future directions for FMT as a therapeutic strategy in neurodegenerative diseases. Across studies, a consistent trend is the modulation of gut microbiota composition, which has been associated with reduced neuroinflammation and improved clinical outcomes in conditions such as PD, AD, and MS. Despite these promising findings, significant challenges persist, including variability in donor selection, inconsistent microbial engraftment, and the absence of standardized treatment protocols. While many studies report positive effects, inconsistencies in efficacy, especially in clinical trials, highlight the complexity of gut–brain interactions and the necessity for deeper mechanistic exploration. Future efforts should focus on refining FMT through personalized strategies, such as microbiota profiling, dietary interventions, and the development of engineered probiotics, to improve therapeutic results and address existing difficulties related to long-term stability and safety. Achieving a more thorough understanding of the underlying mechanisms and advancing controlled clinical trials are critical steps toward establishing FMT as a dependable intervention for neurodegenerative conditions.

## 3. Mechanistic Insights: How FMT Modulates the Gut–Brain Axis

Gut dysbiosis plays a significant role in the development of neurodegenerative diseases, with FMT showing promise in reestablishing microbial equilibrium. A systematic review encompassing 42 studies identified *Bacteroidetes*, *Firmicutes*, and Proteobacteria as dominant phyla across both healthy and disease-affected groups. In PD, increased levels of *Akkermansia*, *Verrucomicrobiaceae*, *Lachnospiraceae*, and *Bifidobacterium* were observed, while AD was linked to *Bacteroides* and *Acidobacteriota* [61]. Another review corroborated the connection of *Akkermansia*, *Bifidobacterium*, and *Faecalibacterium* with PD, while *Alistipes*, *Bacteroides*, *Bifidobacterium*, and *Blautia* were associated with AD. *Escherichia*/*Shigella* emerged as the most consistently linked microbial group. In a study involving 307 MS patients, stable associations with 11 microbial genera were identified, whereas no consistent microbial links were found for amyotrophic lateral sclerosis (ALS) [62]. Certain microorganisms play a crucial role in disease progression by disrupting the balance of inflammation, modulating neurotransmitter levels, altering gut permeability, and influencing immune responses. Both preclinical and clinical studies underscore their therapeutic potential. In models of dextran sulfate sodium (DSS)-induced colitis, *Akkermansia muciniphila* (AKK) demonstrated protective effects by decreasing inflammatory cytokines (TNF-α, IL-1β, IL-6) and inhibiting NLRP3 inflammasome activation. It also helped restore tight junction proteins (ZO-1, occludin, claudin-1), thereby preserving gut barrier integrity. Another study associated higher AKK levels with enhanced antioxidant enzyme activity (CAT, T-AOC), the increased production of acetate and butyrate, and reduced plasma IL-8 levels, further emphasizing its anti-inflammatory properties [63,64].

Several species of *Bifidobacterium* have been shown to exert beneficial effects on inflammatory regulation, neurotransmitter production, immune modulation, and gut barrier integrity. These findings emphasize the potential therapeutic role of these microorganisms, particularly in the context of gut and systemic health. In an animal study, *Bifidobacterium dentium* was demonstrated to contribute to the production of gamma-aminobutyric acid (GABA) by decarboxylating glutamate through the action of glutamate decarboxylase (gadB) [65]. Similarly, in a study involving mice with DSS-induced colitis, *Bifidobacterium pseudolongum* was found to reduce the mRNA expression levels of pro-inflammatory cytokines (IL-1β, IL-6, IL-10, and TNF-α). Interestingly, the mRNA levels of Tph1, encoding tryptophan hydroxylase 1 (TPH1), were significantly lower in mice treated with this microorganism [66]. Contrasting findings were reported in another animal study, where *Bifidobacterium dentium* was shown to increase serotonin (5-HT) concentrations by inducing the expression of 5-HT receptor subtypes (2a and 4) and the serotonin transporter. Additionally, fecal acetate, an SCFA, was significantly elevated in these animals [67].

**Figure 2:**
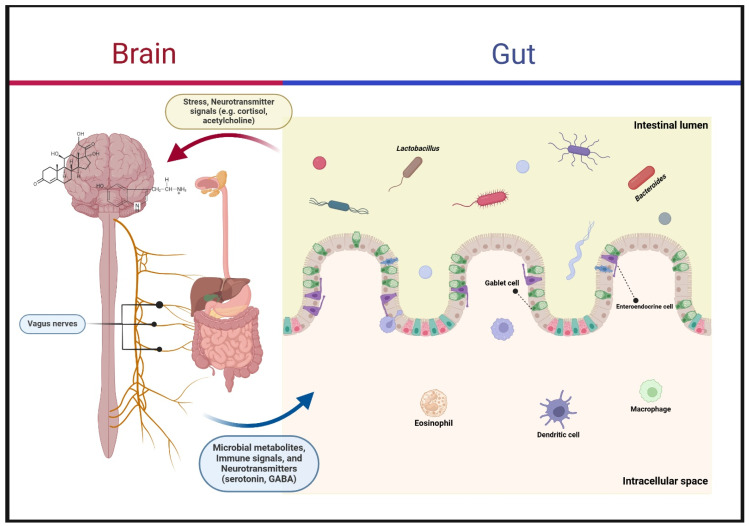
Gut–brain axis. The gut–brain axis represents the bidirectional communication between the central nervous system and the gastrointestinal tract, primarily mediated by the vagus nerve, neurotransmitters, immune signals, and microbial metabolites. The brain regulates gut function through stress hormones (e.g., cortisol) and neurotransmitters (e.g., acetylcholine), influencing digestion and microbiome composition. In return, gut microbes, including *Lactobacillus* and *Bacteroides*, produce metabolites such as serotonin and GABA, which affect brain function, mood, and cognition. Gut-associated immune cells (eosinophil, dendritic cells, macrophages) further modulate this interaction, highlighting the crucial role of gut health in neurological and psychological well-being.

*Bifidobacterium animalis*, through its metabolite lactate, reduced the mRNA levels of inflammatory cytokines (IL-1β, IL-6, IL-10, and TNF-α) in an animal study. This species also decreased the M1/M2 macrophage ratio by interfering with M1 macrophage upregulation. The inhibition of macrophage-induced inflammation was linked to interaction with the TLR4-MyD88 signaling pathway. Moreover, *Bifidobacterium animalis* showed potential in suppressing inflammation associated with NLR family pyrin domain containing 3 (NLRP3) inflammasome signaling [68]. *Bifidobacterium bifidum* was observed to upregulate Foxp3+ regulatory T cells through the interaction of its surface cell polysaccharide, β-glucan/galactan (CSGG), with TLR2. This interaction induced regulatory dendritic cells, highlighting a mechanism for immune modulation [69]. In an in vitro study, *Bifidobacterium infantis* protected epithelial barrier integrity against IL-1β-induced damage by correcting the expression of tight junction proteins, occludin and claudin-1 [70]. A human study involving an infant population revealed key immunological differences based on *bifidobacterial* abundance in the gut microbiota. Infants with low *bifidobacterial* levels exhibited increased neutrophils, basophils, plasmablasts, and memory CD8+ T cells, alongside elevated levels of mucosal-associated invariant T cells. Conversely, infants with *bifidobacteria*-enriched microbiota showed higher numbers of non-classic monocytes, considered anti-inflammatory, and antigen-experienced regulatory T cells expressing the CD39 receptor. These infants also exhibited elevated levels of anti-inflammatory cytokines IL-27 and IL-10 compared to higher levels of pro-inflammatory cytokines IL-17A, IL-13, and IL-1α in the bifidobacterial-deficient group. These findings suggest a critical role for bifidobacteria in shaping immune responses and maintaining homeostasis [71]. The gut–brain axis (Figure 2) and key microorganisms modulated by FMT and their effects on the gut–brain axis are shown in Table 2.

FMT impacts the gut–brain axis by altering the microbial composition, with key players such as *Akkermansia muciniphila*, *Bifidobacterium*, and *Faecalibacterium prausnitzii* supporting gut health, immune function, and neuroprotection. While early findings suggest its potential in addressing neurodegenerative diseases, significant challenges persist, including variability in microbial responses, inconsistent clinical outcomes, and the complexities of selecting suitable donors. Despite these difficulties, FMT offers a promising opportunity for personalized therapeutic approaches. However, further studies are essential to establish standardized protocols, identify precise microbial targets, and ensure its long-term effectiveness in treating neurological disorders.

## 4. FMT: Therapeutic Applications, Challenges, and Ethical Considerations

As previously mentioned, the diversity of the gut microbiome is remarkably high and influenced by a wide range of environmental and genetic factors. To design personalized interventions that yield better outcomes, it is essential to build upon prior research exploring microbiome diversity, functional profiles, microbial interactions, co-occurrence patterns, and the proportional balance of microbiota across different races and regions targeted for intervention. FMT continues to hold significant potential as a therapeutic approach. One promising avenue involves defining the core microbiome of each region, which can be approached in at least four distinct ways, each with its own advantages and limitations:

1. Community Composition: Identifying the most shared microbial taxa within a specific region, which provides a straightforward representation of commonly present species but may overlook functional or ecological dynamics.

2. Functional Profile: Defining the core microbiome based on shared functional capabilities across microbial communities. While this approach emphasizes functional consistency, it may disregard taxa-specific contributions.

3. Ecological Parameters: Incorporating metrics such as taxonomic abundance, interactions, co-occurrence patterns, and other community-level characteristics. Although this provides a holistic view, no standard methods are available.

4. Stability: Examining the factors that contribute to the stability and resilience of microbial communities under environmental and physiological fluctuations. This approach focuses on long-term ecosystem sustainability, but like previous approaches, there are no widely accepted methods for stability evaluation. Such an integrative approach could provide a deeper understanding of regional microbiome characteristics, paving the way for more effective and tailored microbiome-based interventions [85].

FMT is a procedure that alters the recipient’s intestinal tract microbiota by introducing a fecal solution derived from a healthy donor. There are three types of fecal matter preparation used in FMT: fresh, frozen, and lyophilized. Fresh FMT involves the immediate transfer of fecal matter from a healthy donor, demonstrating a 93% efficacy rate in treating *Clostridium difficile* infections. Frozen FMT allows for the storage of donor fecal material for later use, with an efficacy rate of 88% in treating *Clostridium difficile*. Lyophilized FMT involves the dehydration of fecal matter, enabling long-term storage without the need for freezing, and has shown an efficacy rate of 83% in treating *Clostridium difficile* infections [86]. Donors are carefully selected and screened to ensure safety; they must have no personal medical history of autoimmune disease or significant allergy, metabolic disease (like diabetes, metabolic syndrome), gastrointestinal malignancy (and family medical history of the previous three categories), exposure to antibiotics in the prior 3 months (or other drugs which could affect gut microbiota), or recent travel with exposure to epidemic diarrheal disease and they must undergo testing to rule out potential pathogens [87,88,89]. In this context, standardized blood and stool screening tests have been established for the safe application of FMT, though mild variations exist across protocols.

Stool Testing: (1) Bacteria: Screening typically includes *Clostridium difficile*, *Campylobacter*, *Helicobacter pylori* (for the oral administration of FMT), *Salmonella*, Shiga toxin-producing *Escherichia coli*, and *Shigella*. Additional considerations include testing for *Aeromonas*, *Plesiomonas*, *Listeria monocytogenes*, *Yersinia*, *Vibrio cholerae*, and *Vibrio parahaemolyticus*. (2) Viruses: Key pathogens screened include *Rotavirus* and *Norovirus*. (3) Parasites: Tests are performed for *Cryptosporidium*, *Cyclospora*, *Isospora*, and *Giardia*.

Blood Testing: (1) Bacteria: *Treponema pallidum* (syphilis) is screened to prevent transmission risks. (2) Viruses: Comprehensive testing includes *Hepatitis A*, *B*, *C*, and Human Immunodeficiency Virus (HIV). These screening protocols are essential to mitigate the risks of pathogen transmission and ensure donor safety, particularly given the experimental nature of FMT in emerging indications such as neurological disorders [88].

Also, the following social factors should be considered: high-risk sexual behaviors, drug use, incarceration or long-term care facility residence, and body piercing or tattoos in the prior 6 months [88]. According to two systematic review studies, there was no significant evidence to suggest that related donors are superior to unrelated donors in terms of clinical outcomes [90,91]. The fecal solution, prepared by mixing stool with sterile water or normal saline followed by filtration to remove solid particles, can be administered through various routes, including nasogastric or nasojejunal tubes, esophagogastroduodenoscopy, colonoscopy, or retention enema. These methods allow the precise delivery of the microbiota into the gastrointestinal tract, making FMT a promising therapeutic approach for a range of microbiome-associated conditions [87]. FMT is not without potential adverse effects, which can manifest in both the short and long term. The short-term adverse effects of FMT are generally mild to moderate but can occasionally become severe. Commonly reported symptoms include gastrointestinal disturbances such as bloating, abdominal spasms, gaseousness, diarrhea, irregular bowel habits, and abdominal pain or tenderness. Other complications may include constipation, nausea, and belching. In more severe cases, patients have reported fever, hematochezia, and the aggravation of pre-existing inflammatory bowel disease (IBD). Rare but serious adverse events include Gram-negative bacteremia, bowel perforation, and even death in isolated instances. Such severe complications highlight the importance of rigorous donor screening and careful procedural execution. While data on long-term effects are still emerging, there is growing concern about the potential for FMT to influence host metabolism and immune function. Notable long-term effects include an increased risk of obesity and immune-mediated disorders, such as immune thrombocytopenia, rheumatoid arthritis, IBD, and irritable bowel syndrome (IBS). These findings underscore the complex and poorly understood interactions between the transplanted microbiota and the host immune system, warranting further investigation [92]. *Enteropathogenic Escherichia coli* (EPEC) and Shiga toxin-producing *Escherichia coli* (STEC) are potential pathogens that may emerge following an FMT procedure [93].

## 5. Future Directions and Potential Innovations

The bottom-up approach in designing personalized microbiota is an emerging method that can be categorized into two distinct strategies: phenotypic and target-based discovery. The phenotypic approach involves initially testing specific organisms in in vitro settings, followed by evaluating their effects in ex vivo models and animal studies. This method examines various outcomes, including immune, neuronal, metabolic, or microbial responses to the intervention. A prominent example of this approach is *Lactobacillus rhamnosus* JB-1. Studies have demonstrated its ability to reduce corticosteroid release, modulate the central expression of GABA receptors, and improve stress-related social and exploratory behaviors in mice [94]. The target-based approach leverages in silico predictions to evaluate the probiotic capacity of microorganisms to produce molecular effectors with potential modulatory roles in host or microbial pathways. This method necessitates the integration of multi-omics technologies, including genomics, transcriptomics, metabolomics, and proteomics, to comprehensively analyze the relevant mechanisms. A notable example of this strategy is the characterization of the *Hafnia alvei* 4597 strain, selected for its α-melanocyte-stimulating hormone (α-MSH) mimicking effect. This mimicry underpins its anorexigenic potential, which has been demonstrated in both murine and human studies [94].

Synthetic microbial communities (SynComs) are carefully curated consortia of microorganisms, often isolated from human mucosa or feces, designed to achieve specific therapeutic effects. These communities typically consist of two or more microbial species working synergistically to modulate host–microbiota interactions [95]. For instance, an in vivo study compared two live biotherapeutic products: GUT-103, a consortium of 17 microbial strains that demonstrated protective effects in IBD by preventing chronic immune-mediated colitis, and GUT-108, which effectively reversed chronic T-cell colitis in a humanized mouse model. The study highlighted the potential of GUT-108 not only for IBD but also for other conditions characterized by disrupted intestinal permeability and dysbiosis. These findings underscore the promise of SynComs as next-generation microbiota-based therapies, though further research is needed to translate these preclinical successes into safe and effective clinical applications [96].

Numerous studies have explored the integration of FMT with complementary interventions, such as dietary modifications, exercise, and pharmacological treatments, to enhance its efficacy. A scoping review highlighted the supportive role of diet in influencing compositional, functional, and clinical FMT outcomes. However, the available data remain limited and heterogeneous, with inadequate details regarding dietary protocols and their mechanisms of action [97]. Zinc supplementation prior to FMT has shown promise in patients with recurrent *Clostridioides difficile* infection (rCDI), likely due to its ability to address the high prevalence of malnutrition in this population [98]. Additionally, a randomized controlled trial (RCT) demonstrated that adherence to a Mediterranean diet reduced homeostasis model assessment-estimated insulin resistance (HOMA-IR) and lipid levels. However, when combined with donor FMT, no substantial synergistic effects were observed [99]. Another study compared two groups receiving FMT, where the second group’s donors were placed on a pre-diet regimen prior to FMT intervention. Recipients in this group exhibited a significant microbiota shift toward the donor’s composition, particularly with an increase in *Eubacterium_sp_AF228LB*. Functionally, this shift was accompanied by an enrichment of branched-chain amino acids, suggesting a diet-mediated enhancement in microbial metabolic activity [100].

These findings underscore the potential of combining FMT with tailored dietary interventions to optimize therapeutic outcomes. However, the lack of standardized methodologies and detailed mechanistic studies highlights the need for further research to establish evidence-based guidelines for such integrative approaches. Exercise, as a non-invasive intervention, holds significant promise in modulating gut microbiota composition, functional capacity, and metabolic output. Evidence suggests that physical activity can elicit beneficial changes in gut microbiota, although its effects may vary based on individual factors such as body composition. In a human study, sedentary individuals underwent six weeks of endurance-based exercise training, followed by a six-week sedentary washout period. The findings revealed that exercise increased fecal SCFA concentrations, particularly butyrate, in lean participants. This was accompanied by an enrichment of butyrate-producing bacterial taxa, including *Clostridiales* spp., *Lachnospira* spp., *Roseburia* spp., *f. Lachnospiraceae*, and *Faecalibacterium* spp. However, these beneficial microbial shifts were not observed in obese participants, suggesting that individual physiological differences may mediate exercise-induced microbiota changes [101].

In a clinical study involving melanoma patients resistant to anti-PD-1 immunotherapy, FMT was employed as a complementary intervention. Post-FMT analyses revealed the increased activation of CD8+ T-cells, a key component of antitumor immunity, alongside a reduction in interleukin-8 expression from myeloid cells, which is often associated with an immunosuppressive tumor microenvironment. These results underscore the potential of FMT to overcome resistance to immune checkpoint inhibitors by reprogramming the gut–immune axis [102]. These findings highlight the promising role of integrating pharmacological treatments with microbiota-based strategies to optimize therapeutic responses. Further research is needed to elucidate the underlying mechanisms and to explore their clinical applicability across a broader spectrum of diseases. Artificial intelligence (AI) holds transformative potential in advancing FMT research and applications, offering capabilities far beyond traditional methods. However, significant challenges remain, including data limitations, a lack of algorithm transparency, and ethical considerations. Despite these hurdles, AI’s anticipated roles in FMT include response prediction and optimization through donor, recipient, and microbiome-specific approaches. In a noteworthy study, Shtossel et al. proposed an AI-driven algorithm designed to identify optimal FMT donors and predict recipient outcomes based on donor microbiota profiles. The algorithm also facilitates FMT optimization to achieve desired therapeutic effects. This innovation is particularly valuable in cases where there are substantial differences between the microbiota of the donor and the post-transplant recipient. Their findings demonstrated that FMT could be effectively performed using either an ideal donor phenotype or a cultured microbial consortia, with promising results in both human and murine models [103].

**Figure 3:**
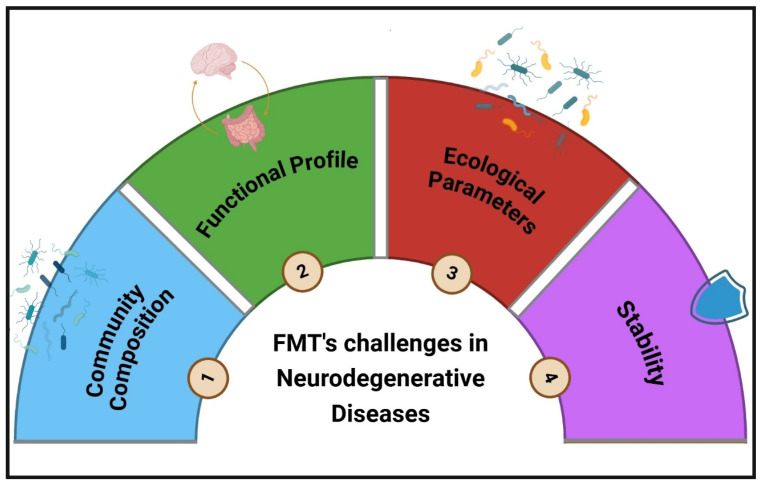
Challenges of FMT in neurodegenerative diseases. FMT holds promise as a therapeutic approach for neurological disorders by modulating gut microbiota, but several challenges hinder its clinical application. These include (1) community composition, referring to the variability in donor and recipient microbiomes; (2) functional profile, which involves the ability of transplanted microbiota to restore key metabolic and immune functions; (3) ecological parameters, such as competition between transplanted and resident microbes; and (4) stability, which relates to the long-term persistence of transplanted microbiota in the host. Addressing these factors is essential for optimizing FMT as a viable treatment for neurodegenerative conditions.

These advancements underline the ability of AI to bridge computational and clinical sciences, paving the way for personalized FMT strategies tailored to specific disease phenotypes. Despite challenges related to data availability, algorithm transparency, and ethical considerations, the integration of AI into FMT research marks a pivotal step toward precision medicine, promising improved therapeutic outcomes and a deeper understanding of the complex interplay between microbiota and health. Large-scale clinical trials are essential to validate the safety and efficacy of the various innovative approaches discussed. Future research should prioritize developing a comprehensive framework to define regional core microbiomes based on functional, ecological, and stability metrics. These metrics could inform the selection of optimal donors or the design of synthetic FMT interventions tailored to specific conditions, including neurodegenerative disorders like PD and AD disease. Additionally, advancing therapeutic precision through engineered probiotics, such as those designed to target specific neurotransmitter pathways or systemic inflammation, represents a promising avenue. Proposing SynComs engineered to produce neurotransmitter precursors (serotonin, GABA) or to modulate inflammatory responses further highlights the untapped potential of microbiota-based interventions. These directions not only offer significant prospects for enhancing FMT efficacy but also open new horizons for addressing complex, multifactorial diseases with precision medicine approaches (Figure 3). Alternative strategies for FMT for gut microbiota modulation are shown in Table 3.

## 6. Conclusions

FMT has gained attention as a promising therapy for neurodegenerative disorders by addressing gut dysbiosis and influencing the gut–brain axis. Both preclinical and clinical evidence indicate that FMT can help restore microbial equilibrium, reduce neuroinflammation, and enhance cognitive and motor functions in conditions like PD, AD, MS, and ALS. Despite these encouraging findings, several challenges need to be addressed, including variations in donor selection, inconsistent clinical results, and a limited understanding of the long-term consequences. For FMT to become a viable treatment option, future research should prioritize standardizing procedures, refining donor screening methods, and incorporating microbiome-based precision medicine. Combining FMT with complementary approaches such as dietary modifications, pharmacological therapies, and engineered probiotics could further enhance its effectiveness. Although FMT has yet to be established as a standard treatment for neurodegenerative diseases, its potential to target underlying pathological processes underscores the importance of further exploration through rigorously designed clinical trials.

## Figures and Tables

**Table 1:** FMT in neurodegenerative diseases: models, mechanisms, and outcomes.

Purpose of FMT Treatment	Biological Objects Used	Microbiota Changes	Mechanistic Insights	Effect of FMT Treatment	Reference Number
Alzheimer’s Disease (AD)	APPswe/PS1dE9 transgenic (Tg) mice	↑*Bifidobacterium*, ↑*Lactobacillus*	↓IL-1β, ↓IL-6, ↓APP, ↓BACE1	Improved cognitive deficits, ↓Aβ plaque deposition, ↑neuronal regeneration	[54,55]
	Aged mice	↑SCFA-producing bacteria	Anti-inflammatory effects	↓amyloid plaques, ↑memory, ↓anxiety	
	Mice receiving FMT from methionine-restricted (MR) diet mice	↑*Roseburia*, ↑*Blautia*, ↓*Anaerotruncus*	↓TNF-α, ↓IL-1β	↑SCFAs, ↑cognitive function	
Multiple Sclerosis (MS)	Human patients	↑*Phascolarctobacterium*, ↑*Hungatella*	↑Intestinal barrier integrity	Safe with mild gastrointestinal discomfort, altered microbiota composition, ↑gut barrier function	[56]
	EAE mice	↓*Akkermansia*, ↑*Bacteroidetes*, ↑*Prevotella*	↓Microglial activation, ↑MBP, ↓NF-L	↓neuroinflammation, ↑blood-brain barrier integrity	
Parkinson’s Disease (PD)	Rotenone-induced PD mice	↓*Desulfovibrio*, ↓*Akkermansia*	↓TLR4/MyD88/NF-κB signaling	↓neuroinflammation in substantia nigra, ↑motor function	[57]
	MPTP-induced PD mice	↑*Firmicutes*, ↓*Proteobacteria*	↑Dopamine, ↑5-HT, ↓TLR4/TBK1/NF-κB/TNF-α signaling	↑motor function, ↓microglial activation	[58]
	Human patients	↑*Blautia*, ↑*Roseburia*	Microbiota–gut–brain axis modulation	Improved MDS-UPDRS motor scores, ↑gut microbiota diversity	[59]
	Human patients in a clinical trial	↑*Roseburia*, ↑*Collinsella*, ↓*Proteobacteria*	↑SCFAs, ↓Inflammation	Improved constipation, sleep quality, ↓falls, temporary motor improvement	[60]

↑: increase; ↓: decrease.

**Table 2:** Key microorganisms modulated by FMT and their effects on the gut–brain axis.

Microorganism	Main Effects on the Gut–Brain Axis	Mechanistic Pathways	Relevance to Neurodegenerative Diseases	Ref.
*Akkermansia muciniphila*	↑gut barrier integrity, ↓inflammation	Upregulates tight junction proteins (ZO-1, occludin), ↑acetate and butyrate levels, ↑anti-inflammatory M2 macrophages	Associated with improved gut barrier function in MS, PD, and AD	[72]
*Bacteroides*	SCFA production, immune modulation	Produces butyrate and propionate, interacts with TLR signaling to regulate immune responses	↑in AD, modulates inflammation and neurotransmitter metabolism	[73]
*Bifidobacterium* (*B. longum*, *B. bifidum*, *B. pseudolongum*)	↑regulatory T cells, modulates neurotransmitter synthesis	Produces GABA and serotonin precursors, ↑IL-10 secretion, regulates tryptophan metabolism	Linked to PD, AD, and MS; associated with improved cognitive and motor function	[74]
*Faecalibacterium prausnitzii*	Anti-inflammatory effects, ↑gut homeostasis	Produces butyrate, inhibits NF-κB and NLRP3 inflammasome activation, ↑Treg cells	↑post-FMT, linked to ↓neuroinflammation in PD and AD	[75]
*Prevotella*	↑SCFA production, supports gut–immune balance	↑production of propionate and butyrate, modulates gut permeability	↑post-FMT, beneficial for immune regulation in MS and PD	[76]
*Escherichia/Shigella*	Pro-inflammatory, linked to gut dysbiosis	Activates TLR4/MyD88/NF-κB pathway, ↑systemic inflammation	Associated with AD pathology, linked to BBB disruption	[77]
*Roseburia*	↑butyrate production, supports gut barrier integrity	Produces butyrate, upregulates tight junction proteins, ↓oxidative stress	Linked to improved cognitive function in AD models	[78]
*Lactobacillus* (*L. rhamnosus*, *L. reuteri*)	Modulates neurotransmitter production, ↓anxiety and depression	↑serotonin and GABA production, regulates vagus nerve signaling	Protective in stress-related neurodegeneration, potential benefits in PD and AD	[79]
*Alistipes*	Modulates tryptophan metabolism, anti-inflammatory effects	↑kynurenic acid production, ↓neurotoxic quinolinic acid	Associated with cognitive benefits in AD models	[80]
*Blautia*	Supports metabolic homeostasis, ↓inflammation	Produces SCFAs, interacts with bile acid metabolism	↑after FMT in PD, linked to gut–brain homeostasis	[81]
*Clostridium cluster XIVa*	↑regulatory T cell differentiation, ↑gut integrity	Produces butyrate, induces TGF-β signaling, supports IL-10 secretion	Protective in MS, supports gut–immune balance	[82]
*Desulfovibrio*	Pro-inflammatory, linked to gut dysbiosis	Produces hydrogen sulfide (H2S), disrupts gut barrier integrity, ↑LPS translocation	↑in PD, associated with worsened motor symptoms	[83]
*Ruminococcus*	Modulates bile acid metabolism, ↑gut integrity	Produces secondary bile acids, supports tight junction proteins	Linked to improved metabolic and neurological outcomes	[84]

↑: increase; ↓: decrease.

**Table 3:** Alternative strategies for FMT for gut microbiota modulation.

Strategy	Mechanism of Action	Potential Applications	Challenges and Limitations	Ref.
Engineered *Escherichia coli* Nissle 1917	Genetically modified to deliver therapeutic molecules and modulate immune responses	Inflammatory bowel disease (IBD), metabolic disorders, gut–brain axis modulation	Safety concerns, potential horizontal gene transfer	[104]
Synthetic Microbial Consortia (SynComs)	Designed communities of beneficial microbes to restore gut homeostasis	Neurodegenerative diseases, IBD, metabolic disorders	Standardization issues, ecological stability, regulatory approval	[105]
Postbiotics	Metabolites or cell components from probiotics with bioactive properties	Modulating inflammation, immune function, gut barrier integrity	Limited clinical validation, variability in effectiveness	[106]
Phage Therapy	Bacteriophages target specific pathogenic gut bacteria to reshape microbiota composition	Antibiotic-resistant infections, microbiota dysbiosis	Specificity challenges, safety concerns	[107]
Prebiotics	Dietary fibers and compounds that selectively promote beneficial gut microbes	Supporting gut–brain health, metabolic balance, immune modulation	Individual variability, limited control over microbial composition	[108]
Enterohormone-Based Therapies	Modulating gut hormone levels to influence microbiota composition and function	Gut–brain axis disorders, metabolic diseases	Complexity in hormone–microbiota interactions, long-term effects unknown	[109]
